# Malaria

**Published:** 1898-02

**Authors:** R. L. Denman

**Affiliations:** Durst, Texas


For the Texas Medical Journal.

### MALARIA.

BY R. L. DENMAN, M. D., DURST, TEXAS.

There is now in progress one of those revivals of interest in, and discussion of malaria and its protean manifestations, which periodically sweep over the country; the journals are full of articles on the subject. It has been discussed through the press and medical societies until the subject is almost threadbare, yet it is full of interest still. Each writer sees, or thinks he sees, something not observed by anyone else, and forthwith has his say on the subject. It is true, these discussions have stimulated investigation, and it is counted amongst the greatest advances of modern medicine to know at last the real cause of "malaria;" that it is a germ or organism, technically, *plasmodium malariæ*; but we are far from knowing the exact cause or mode of propagation and development of the germ itself. It is universally ascribed to the action of heat and moisture on vegetable matter. But whether this action results in the direct production of the organism, or produces another form of organism which, entering the blood, is there developed into the form in which we see it, we do not know. Certain it is that no eye hath seen the *plasmodium malariæ* outside of the blood of man (one writer claims that it is also found in the blood of pigeons). I say, it is a great advance on the state of medical science when Summerville's *algæ spores* were accepted as the cause of the chill and fever, or when the disease was thought to be produced by "bad air," the belief that gave to it its specific name—*mal-aria.*

Nor is the question yet determined, altho' it has been discussed in season and out of season, by every writer, whether the germ is air-borne or water-borne, strong arguments being adduced in support of both claims. It is highly probable that, like the shield, which to one knight was golden, and to the other, who viewed it from the opposite standpoint, it was silver, whereas it was both golden and argent, malaria is communicated by both methods; by drinking water and by breathing an infected atmosphere.

At any rate malaria, as we see and understand it, is the cause of more trouble to the doctor than almost any other one thing, for in some sections of the country, the "malarial belt," no matter what else a patient may have, he has malaria also. It complicates most diseases that we meet in this country. So well is this known, that quinine is given for almost everything; for everything at least that is characterized by anything approaching periodicity. Our lying-in women very often develop remittent fever, or chill and fever, though they may have been up and about previous to confinement. Dr. Leake tells of a case (TEXAS MEDICAL JOURNAL) of malarial irritation of the neck of the bladder. Neuralgia, especially of the face, is often made obstinate by the existence of malaria in the system, and will yield to nothing but quinine. With persons residing in a malarial section, there is something of a tolerance of the poison established; the system is charged with it; but it is exhaled or thrown off, and does not accumulate in sufficient quantity to produce an explosion, until, or unless something occurs that will weaken the vital powers or arrest the process of elimination, when an attack of chill and fever or of remittent fever is precipitated. To allow the bowels to become obstinately constipated will sometimes do this. I have known a wound or injury to do so. I knew a hale old lady of eighty who was attacked with chill and fever after having fallen and sprained her wrist. Since the discovery of the plasmodium only has the action of quinine in malaria been understood. Since the days of Peruvian bark, before the alkaloid quinia had been separated and extracted, though known to be the active principle, it has been known that quinia will cure the common forms of malarial disease, but it was entirely empirical; no one had any idea of its *modus operandi.* Its action is now universally known; it destroys the plasmodium *in situ;* it is demonstrable under the microscope, and so well is it now understood that quinine is thus used for diagnostic purposes, for differentiation of fevers, and is absolutely unfailing.

I have been led to the foregoing remarks and to write this paper partly by reading the papers of Drs. Brodnax and Briggs on the subject of malaria, published in the TEXAS MEDICAL JOURNAL. They dispute the efficacy of quinine at this late day. In light of the knowledge of the specific action of quinine on the organism known to be characteristic of malaria, and the invariable and only cause, it looks like a stultification to say, as Dr. Brodnax does, "that quinine is uselesss in the condition known and recognized as malaria," and the actual denial of the existence of malaria; but asserts that what we see and recognize as the manifestation of the malarial poison, in the system is caused by "filth in the bowels!" It would be a worse stultification were I to attempt, at this late date, to refute such absurd doctrine. If the accumulated revelations at the bedside recorded in thousands of text-books, journals and essays, by men of eminence in the profession; if years of clinical experience have not demonstrated to the doctor that the salts of quinine are specific for malaria; if demonstration of its mode of action, its destructive effect upon the germ known to cause these manifestations have not convinced Dr. Brodnax that he is wrong, nothing I could say would have any weight with him. There is not a practitioner of any experience who could not testify to the curative effects of quinine in these cases.

I have had nine years practical experience in the treatment of malaria, and thousands of cases, from the most benign to the most malignant and complex forms, and I have run the range of therapeutic resources, but I never yet succeeded in warding off of malarial paroxysm with anything except quinine in some form. I have persistently tried all substitutes, and once came near losing a valuable patient by so doing.

Dr. Brodnax tells us of "malaria that quinine will not cure," and that "antisepticising the intestinal tract with mercury, and the administration of three to five grains of antifibrin fifteen or twenty minutes before chill time" will cure. I have no knowledge of such. There must be another poison called by them "malaria," or "filth in the bowels," differing from that taught by Osler, Flint, Bartholo, Pepper, Quain, Holt, and hosts of other clinicians, for my experience and observations tally with their teachings.

Dr. Brodnax, in one of his articles, has the—shall I call it presumption, temerity or assurance?—to say that the authors of our text-books never saw a case of malarial poisoning! It would be a waste of time to argue with a man holding such views. I am led by the doctor's remarks to apprehend that he is not skilled in the use of quinine and does not discriminate between the well known *excitant* action of small doses and the *sedative* and *antipyretic* action of large ones; I can readily understand how he would be disappointed if, wishing to produce a sedative effect or an antipyrenic action, he should make the mistake of giving small doses. I have often been called to cases of chill and fever or of simple remittent fever where the family, in the absence of medical advice, had attempted to break it up with what they call "broken doses" of quinine, i. e., one, two, three grains, and found that it was necessary only to give quinine *enough*, and the patient would speedily recover. There is skill required in the use of any remedy, and as much so in giving quinine as anything else. It is often necessary to combine it with other remedies to meet certain indications, or we will be disappointed. If there be "filth in the bowels," common sense would demand that it be voided before we can expect quinine or anything else to exert its curative effect.

With regard to that form of fever called "remittent," that does not respond to quinine, and is by some called "typhoid," my opinion is that the disease, in this section, is purely a malarial remittent, "*aestro autumnal*," a neglected or improperly treated remittent fever. The remissions become shorter and shorter, by reason perhaps of insufficient quinine in the beginning, until there is a continuous fever. In my opinion there is then a lesion formed somewhere which is, in itself, sufficient to keep up the fever, malaria having meantime been suspended.

Quinine does no good after a certain time. I contend that this condition will not obtain if quinine be given in the proper manner, in the proper doses, at the right time. At least that has been my experience. In all cases, after satisfying myself that cinchonism has been produced without amelioration of symptoms, I know that one of two things exists: it is not malaria, or else it is the result of the damage done in the beginning.

In another paper, later, I will tell what I know of hæmaturia, a knowledge gleaned from a close study recently of eighteen cases, as this paper has already exceeded the limit assigned it.

